# [Retracted] Human HLA‑F adjacent transcript 10 promotes the formation of cancer initiating cells and cisplatin resistance in bladder cancer

**DOI:** 10.3892/mmr.2024.13187

**Published:** 2024-02-27

**Authors:** Chen Li, Zhenfan Wang, Ninghan Feng, Jian Dong, Xiaoyan Deng, Yin Yue, Yuehong Guo, Jianquan Hou

Mol Med Rep 18: 308–314, 2018; DOI: 10.3892/mmr.2018.9005

Following the publication of this paper, it was drawn to the Editor's attention by a concerned reader that the cellular morphological data in Fig. 1C, the immunofluorescence data shown in Fig. 1E, and certain of the scratch-wound assay data shown in Fig. 2A were strikingly similar to data appearing in different form in other articles written by different authors at different research institutes that had already been published. Owing to the fact that the contentious data in the above article had already been published prior to its submission to *Molecular Medicine Reports*, the Editor has decided that this paper should be retracted from the Journal. The authors were asked for an explanation to account for these concerns, but the Editorial Office did not receive a reply. The Editor apologizes to the readership for any inconvenience caused.

